# Lung Segmentation with Lightweight Convolutional Attention Residual U-Net

**DOI:** 10.3390/diagnostics15070854

**Published:** 2025-03-27

**Authors:** Meftahul Jannat, Shaikh Afnan Birahim, Mohammad Asif Hasan, Tonmoy Roy, Lubna Sultana, Hasan Sarker, Samia Fairuz, Hanaa A. Abdallah

**Affiliations:** 1 Department of Electronics and Telecommunication Engineering, Rajshahi University of Engineering and Technology, Rajshahi 6204, Bangladesh; mefta.ruet19@gmail.com (M.J.); hasan.ruet.ete@gmail.com (H.S.); samiafairuz07@gmail.com (S.F.); 2 Department of Computer Science and Engineering, Royal University of Dhaka, Dhaka 1208, Bangladesh; afnan.iatb1@gmail.com; 3 Department of Data Analytics and Information Systems, Utah State University, Old Main Hill, Logan, UT 84322, USA; tonmoy.roy@usu.edu; 4 Department of Computer Science and Engineering, International Islamic University Chittagong, Chittagong 4318, Bangladesh; lubnasultana825@gmail.com; 5 Department of Information Technology, College of Computer and Information Sciences, Princess Nourah bint Abdulrahman University, P.O. Box 84428, Riyadh 11671, Saudi Arabia

**Keywords:** lung segmentation, convolutional block attention module, dice loss, lightweight residual U-Net, CXR images

## Abstract

**Background:** Examining chest radiograph images (CXR) is an intricate and time-consuming process, sometimes requiring the identification of many anomalies at the same time. Lung segmentation is key to overcoming this challenge through different deep learning (DL) techniques. Many researchers are working to improve the performance and efficiency of lung segmentation models. This article presents a DL-based approach to accurately identify the lung mask region in CXR images to assist radiologists in recognizing early signs of high-risk lung diseases. **Methods:** This paper proposes a novel technique, Lightweight Residual U-Net, combining the strengths of the convolutional block attention module (CBAM), the Atrous Spatial Pyramid Pooling (ASPP) block, and the attention module, which consists of only 3.24 million trainable parameters. Furthermore, the proposed model has been trained using both the RELU and LeakyReLU activation functions, with LeakyReLU yielding superior performance. The study indicates that the Dice loss function is more effective in achieving better results. **Results**: The proposed model is evaluated on three benchmark datasets: JSRT, SZ, and MC, achieving a Dice score of 98.72%, 97.49%, and 99.08%, respectively, outperforming the state-of-the-art models. **Conclusions:** Using the capabilities of DL and cutting-edge attention processes, the proposed model improves current efforts to enhance lung segmentation for the early identification of many serious lung diseases.

## 1. Introduction

The analysis of CXR images is a complex and labor-intensive task, often necessitating the detection of multiple abnormalities simultaneously. Radiologists must meticulously examine each image to identify and evaluate potential problems, such as pneumonia, lung nodules, fractures, or other pathological conditions. This process requires expertise and meticulous attention to detail, as even minor anomalies can have clinical significance. The use of deep learning (DL) in medical imaging can aid medical experts in performing tests and diagnoses, reducing their workload [1,2]. Lung segmentation raises several issues: (1) Nonpathological changes: age, sex, and heart size all affect the size and structure of the lung; (2) Pathological changes: high-intensity opacities can result from severe lung illness; (3) Foreign body coverage: Patient clothing or medical devices may make the lung field undetectable [3]. To train a machine learning system for disease detection and anomaly identification, this investigation may involve identifying clinically relevant information. Using these computational techniques, physicians can diagnose patients more quickly and accurately, improving patient outcomes and treatment quality. Lung disorders are the leading cause of death for children in many countries, including bronchiolitis, bronchitis, bronchopneumonia, interstitial pneumonia, lobar pneumonia, and pneumothorax [4].

Many studies on lung segmentation primarily utilize images depicting mild lesions or healthy chest radiographs. Consequently, evaluating the efficacy of the lung segmentation model becomes imperative when confronted with more intricate chest radiograph images. Accurate identification of lung fields facilitates subsequent computational analysis of these anatomical regions. The advent of DL has sparked a profound revolution in image segmentation, ushering in many techniques that have markedly improved the accuracy and speed of the segmentation process. One such technique is U-Net, a convolutional neural network explicitly engineered to segment biomedical images. It possesses a U-shaped architecture comprising a contracting path (encoder) and an expansive path (decoder). This unique structure enables U-Net to operate with fewer training images while providing highly accurate segmentation [5]. The UNet++ [6] encoder and decoder subnets are connected by nested, dense skip routes, which are intended to improve medical image segmentation. These improved skip paths help the optimizer’s learning process by narrowing the semantic difference between the encoder’s and decoder’s feature maps. Road image extraction using ResUNet [7], a U-Net architectural variation, has produced state-of-the-art (SOTA) results.

The primary motivation for this research is to enhance the early detection of lung diseases, including pneumonia, tuberculosis, and, most importantly, lung cancer. By providing precise regions of interest, the study aims to assist radiologists in improving the accuracy and efficiency of disease diagnosis. Additionally, it seeks to address existing research gaps in lung segmentation by enhancing model quality while reducing computational complexity. Furthermore, this research focuses on developing a more accurate and robust method for predicting lung segmentation masks from chest X-ray (CXR) images, overcoming challenges such as variable image quality to ensure more reliable diagnostic support.

DL can be a powerful tool for lung segmentation, providing several advantages over traditional image processing techniques. These benefits include enhanced accuracy, robust management of variability in medical images, streamlined automation and efficiency, scalability for extensive datasets, integration capabilities with multimodal data, ongoing enhancement through transfer learning and incremental updates, and strengthened capabilities in clinical decision-making and quantitative analysis. Moreover, DL stimulates innovation and interdisciplinary cooperation, driving advances in medical image analysis and ultimately improving patient care outcomes.

The main contributions of this research can be summarized as follows:

1. A custom Lightweight U-Net model is proposed by combining the strengths of residual paths, CBAM, and ASPP with LeakyReLU activations for feature extraction to handle the various channel and spatial perspectives of CXR images and predict segmentation accurately.
2. The effectiveness of the model was examined on three different popular datasets (JSRT, SZ, and MC), where it outperformed all other SOTA models.
3. A random chest X-ray dataset from Kaggle was used for external validation, demonstrating the model’s effectiveness and robustness.
4. The complexity of the model was analyzed, demonstrating that the proposed model is lighter than all other SOTA models and also more efficient while maintaining high performance.

To effectively extract important features in complex and noisy backgrounds, this work introduces a custom Lightweight U-Net model, integrating Residual Paths, the Convolutional Block Attention Module (CBAM), and Atrous Spatial Pyramid Pooling (ASPP) with LeakyReLU activations. This combination enhances feature extraction by effectively capturing spatial and channel-wise dependencies, leading to superior segmentation performance. Unlike conventional Residual U-Net implementations, the proposed model is designed to be lighter and computationally more efficient, making it suitable for real-time medical applications. Furthermore, the model has been rigorously evaluated on three benchmark datasets (JSRT, SZ, and MC), with external validation conducted on a Kaggle chest X-ray dataset, demonstrating its robustness and generalization ability. The results show that the proposed method outperforms state-of-the-art (SOTA) models while maintaining a lower computational footprint.

The rest of this paper is organized as follows: Section 2 presents a review of related works on lung segmentation techniques, highlighting their strengths and limitations. Section 3 describes an overview of the datasets, preprocessing steps, and the proposed methodology, detailing the architecture of the Lightweight Convolutional Attention Residual U-Net and its key components. Section 4 discusses the results obtained from various datasets, including performance comparisons with state-of-the-art models. Section 5 presents a discussion on the model’s strengths, limitations, and potential clinical applications. Finally, Section 6 concludes the paper and outlines possible directions for future research.

## 2. Literature Review

Lung segmentation in chest X-ray images is a crucial step in medical image analysis, aiding in the early detection and diagnosis of lung diseases. Over the years, researchers have explored various deep learning-based approaches to improve segmentation accuracy, efficiency, and robustness; yet, challenges remain in handling complex cases and diverse imaging conditions. Xu et al. [8] proposed TransCotANet, a novel deep learning architecture for lung segmentation in chest X-ray images, addressing challenges related to lung morphology variations and noise interference. Their model integrates a CotA module within a U-Net framework to enhance global feature aggregation and improve segmentation accuracy. Evaluated on three benchmark datasets—JSRT, Montgomery County, and Shenzhen—TransCotANet achieved Dice similarity coefficients of 99.03%, 98.02%, and 97.66%, respectively, outperforming existing state-of-the-art models. Despite its high accuracy, the model faces challenges such as increased computational complexity, sensitivity to noise, and difficulty in segmenting small target regions, highlighting areas for future optimization.

Akter et al. [9] introduced a Pix2pix GAN-based approach for automating lung segmentation in chest X-ray images, addressing challenges posed by overlapping organs and anatomical variations. Using the Montgomery dataset for training and the Shenzhen dataset for robustness testing, their model achieved a Dice coefficient of 98.05% and an accuracy of 98.25%, outperforming traditional methods. By leveraging adversarial learning, L1 loss optimization, and data augmentation techniques, the model demonstrated enhanced generalization capabilities. However, limitations such as dataset constraints, potential overfitting, and generalization challenges in diverse clinical settings highlight areas for further improvement. Khorasani et al. [10] introduced FAT-Net, a deep learning model for lung segmentation in chest X-ray images, integrating an auxiliary transformer branch to enhance global contextual understanding. Using the Montgomery and Shenzhen datasets, the model achieved an accuracy of 98.12% and a Dice coefficient of 96.10%, outperforming baseline models such as Attention U-Net and SegNet. FAT-Net’s dual encoder–decoder architecture, combined with transformers, improved segmentation quality while maintaining computational efficiency. However, challenges such as generalizability to diverse datasets and edge case performance indicate areas for further refinement in clinical applications.

Hao et al. [11] introduced VAEL-Unet, a lightweight deep learning model for lung segmentation in chest X-ray images, emphasizing computational efficiency and segmentation accuracy. Utilizing the Kaggle chest X-ray dataset and LUNA16, their model incorporated a MobileNetV3-based encoder, a U-Net-inspired decoder, and the Convolutional Block Attention Module (CBAM) to enhance feature extraction. Achieving an accuracy of 97.69%, VAEL-Unet outperformed traditional U-Net and SegNet models while significantly reducing parameter count and training time. However, its performance may slightly lag behind more complex architectures like DeepLabV3Plus_ResNet50 in challenging cases, highlighting the need for further optimizations in diverse imaging conditions. Sulaiman et al. [12] proposed a CNN-based model for lung segmentation in chest X-ray images, incorporating a concatenate block for enhanced feature extraction and a transpose layer to improve spatial resolution. Using a Kaggle-sourced dataset with 5-fold cross-validation, their model achieved a high accuracy of 97%, an IoU of 0.93, and a Dice similarity coefficient of 0.96, outperforming baseline methods. While the model demonstrated robust segmentation capabilities, its performance remains dependent on dataset quality and may require further tuning for broader generalizability across diverse imaging conditions and pathological variations.

Alam et al. [13] proposed AMRU++, an attention-based multi-residual U-Net++ model for lung segmentation in chest X-ray images, particularly for diseased lungs with severe abnormalities. Using a diverse dataset, including Montgomery, JSRT, Shenzhen, GMH, and COVID datasets, their model achieved a Dice similarity coefficient of 0.9363 and outperformed ResUNet++ and I2U-Net. Key innovations include multi-scale residual blocks, attention mechanisms, and a custom augmentation technique (Selective Cutout) to improve segmentation accuracy. However, AMRU++ has a higher computational cost and risks overfitting, requiring careful optimization for broader applications. Gite et al. [14] proposed an enhanced lung segmentation approach for tuberculosis diagnosis using deep learning, comparing U-Net, U-Net++, FCN, and SegNet architectures. Utilizing the Montgomery and Shenzhen datasets, their study demonstrated that U-Net++ outperformed traditional models, achieving an accuracy of 98.74%, a Dice similarity coefficient of 97.96%, and a mean IoU of 95.96%. The improved architecture minimized data leakage and enhanced feature representation, leading to more precise segmentation. However, increased computational requirements and reliance on large datasets pose challenges for deployment in low-resource medical settings.

Zhang et al. [15] introduced the HAFS model, a novel lung image segmentation algorithm that addresses the challenges of occlusion in medical imaging, particularly in chest X-rays. The authors incorporated a hybrid skip connection and attention mechanism into the YOLOv8 framework to enhance feature fusion and preserve critical details. Their approach utilized two publicly available datasets, Montgomery and Shenzhen chest X-rays, and applied data augmentation techniques to improve model performance. The HAFS model achieved significant improvements in segmentation accuracy compared to traditional methods, with enhanced feature representation and reduced computational overhead. However, it faces challenges in handling input variability and requires advanced computational resources for implementation. Yuan et al. [16] introduced a novel pneumothorax segmentation method that integrates anatomical constraints to improve deep learning (DL) models for medical imaging. Their approach leverages anatomical knowledge of the thoracic cavity, using auxiliary lung segmentation datasets to create accurate constraints. The four-phase pipeline includes lung area segmentation, morphological operations to refine segmentations, and a discriminator to filter unreliable constraints before training the final pneumothorax segmenter. Evaluated on the SIIM-ACR pneumothorax dataset, the model demonstrated performance improvements over baseline architectures, excelling in metrics such as IoU, Dice similarity coefficient, and Hausdorff distance. However, the model is sensitive to hyperparameters and morphological choices, and may struggle with anatomical variations or atypical pneumothorax presentations.

Dathar Abas Hasan et al. [17] proposed a lung segmentation method for chest X-ray images using the Deeplabv3plus CNN model, aiming to improve segmentation accuracy, particularly in regions with ambiguous borders due to overlapping anatomical structures. They used the Shenzhen dataset, which consists of 662 chest X-ray images, and applied various augmentation techniques to enhance the training data. The Deeplabv3plus model, utilizing Atrous Convolution and Atrous Spatial Pyramid Pooling (ASPP), demonstrated high accuracy, achieving a global accuracy of 97.42%, a Jaccard index of 93.49%, and a Dice similarity coefficient of 96.63%. While the model showed superior performance compared to existing methods, its reliance on a single dataset and computational resource requirements limits its generalizability and practical implementation.

Turk and Kılıçaslan [18] presented a study titled “Lung Image Segmentation with Improved U-Net, V-Net, and Seg-Net Techniques”, focusing on enhancing tuberculosis detection through advanced segmentation models. They utilized two public datasets, Montgomery and Shenzhen, combining them to create a dataset of 800 chest X-ray images, split into 640 for training and 160 for testing. The authors introduced enhanced versions of U-Net, V-Net, and Seg-Net, integrating attention mechanisms, residual connections, and non-local blocks to improve segmentation accuracy. Their results demonstrated that the improved U-Net and V-Net outperformed traditional methods, with Dice similarity coefficients of 96.43% and 96.42%, respectively. While the models showed significant improvements in accuracy, they also faced challenges with longer training times and potential overfitting in small or noisy datasets. The first author of the paper is Fuat Turk. 

Xu et al. [19] introduced an advanced lung segmentation model using a Kiu-Net-based multi-interaction feature fusion network to overcome the limitations of traditional U-Net variants in detecting smaller lung structures and segmenting boundaries. Their approach integrates Kiu-Net and U-Net with specialized modules such as the Cross-Residual Fusion Block and Global Information Module, which enhance feature extraction and segmentation accuracy. The model demonstrated impressive results, achieving Dice similarity coefficients (DSCs) of 99.2% on the Montgomery County dataset and 98.3% on the Shenzhen dataset. However, the complexity of the model may lead to increased computational demands and challenges in handling images with heavy noise or artifacts.

Ji et al. [20] introduced the ResDSda_U-Net, a novel enhancement of the traditional U-Net architecture, specifically designed for the automatic segmentation of pulmonary nodules in lung CT images. Their approach integrates a Depthwise Over-Parameterized Convolutional layer, a denser Dense Atrous Spatial Pyramid Pooling module, and attention mechanisms to improve feature extraction and segmentation accuracy. The model outperformed several existing networks, achieving a high Dice similarity coefficient of 86.65% and an intersection over union of 76.73%. Despite its impressive accuracy, the model requires significant computational resources and may struggle with smaller nodules in complex CT images. This work significantly contributes to advancing medical image segmentation, offering valuable support for the early diagnosis and treatment of lung cancer. 

Khehrah et al. [21] presented a fully automated computer-aided detection (CAD) system for lung nodule detection in CT images, eliminating the need for manual segmentation by radiologists. Using the Lung Image Database Consortium (LIDC) dataset, which consists of 70 CT scan cases with 250–350 images per scan, the authors developed a multi-phase pipeline involving histogram-based thresholding for lung segmentation, statistical and shape-based feature extraction, and classification using a support vector machine (SVM). The proposed method achieved a high sensitivity of 93.75%, outperforming several existing approaches. Its advantages include being lightweight, explainable, and implementable on simple computers, making it a cost-effective solution. However, the study also has limitations, such as a relatively small dataset as well as very low accuracy, reliance on predefined shape-based rules that may not generalize well to complex cases, and a lack of deep learning integration, which has shown promising results in recent research. Additionally, while the system effectively distinguishes nodules from non-nodules, it still produces false positives, with false positive per image (FPI) and false positive per exam (FPE) values of 0.13 and 0.22, respectively.

Despite significant advancements in lung segmentation using deep learning techniques, several research gaps remain. Many existing models struggle with high computational complexity, limiting their real-time clinical applicability. Additionally, most studies focus on datasets with mild abnormalities, leading to reduced generalizability in complex and diverse pathological cases. The performance of segmentation models is often dataset-dependent, highlighting the need for robust models that can generalize across different imaging conditions. Furthermore, external validation using different datasets is also rare. Addressing these gaps will be crucial for developing more efficient and clinically viable lung segmentation models.

## 3. Proposed Method

**Figure 1:**
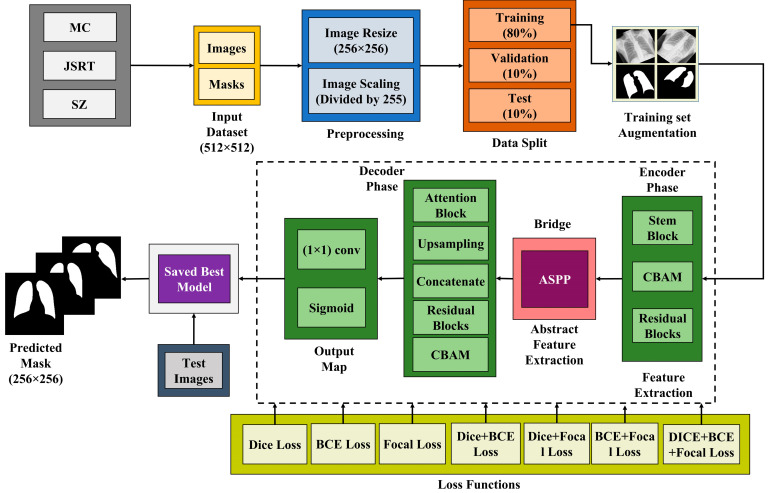
Proposed workflow for lung segmentation.

Figure 1 illustrates the proposed workflow for segmenting lung images. It starts by pre-processing the input datasets by resizing and scaling the images. The images are resized to 256 × 256 pixels and then normalized by dividing each pixel by 255. Then the datasets are split into three parts: training (80%), validation (10%), and testing (10%) sets. The proposed model performs feature extraction using a four-step process. Firstly, the images undergo the encoder phase, or contraction path, where they capture the contextual information and extract features from the input images. Subsequently, they traverse the most profound segment of the network, known as the bottleneck layer, which serves as the intermediary between the encoder and decoder. The ASPP module is utilized as the limiting layer. Before the upsampling process, it captures the most abstract characteristics of the incoming image. Subsequently, the decoder path, also known as the expanding path, reconstructs the spatial dimension and enhances the segmentation. Ultimately, the last layer generates the segmentation map by decreasing the depth of the feature maps to match the number of classes. The proposed model was trained using several types of loss functions to achieve optimal results. The best-performing model was saved and used to predict the masks of the test images. This workflow demonstrates a comprehensive approach to the segmentation of the CXR images with an improved residual U-Net that integrates a CBAM module. The step-by-step process of lung segmentation using the Lightweight Residual U-Net is outlined in Algorithm 1, providing a structured overview of the methodology.
**Algorithm 1:** Lung Segmentation using Lightweight Residual U-Net1: **Input:** CXR images, mask images. 2: Preprocess the data by resizing the images to 256 × 256 pixels and normalizing the pixel values between 0 to 1 by dividing each pixel by 255. 3: Split the images into training, validation, and testing sets. 4: Augment the training set using the different techniques mentioned in the study. 5: Select Dice loss as the primary loss function with the Adam optimizer, train the lightweight segmentation model, and save the best-performing model based on validation loss. 6: **Output:** Predict the results on the test set.

### 3.1. Dataset Description

Three publicly accessible CXR datasets are used to thoroughly assess the proposed model’s lung segmentation performance: the Shenzhen (SZ) dataset [22], the Montgomery County (MC) dataset [23], and the Japanese Society of Radiological Technology (JSRT) dataset [24]. The JSRT dataset consists of a total of 247 CXR images at 2048 × 2048 pixels, which include 90 images of normal, healthy lungs, while the other images show different abnormalities. The SZ dataset contains 662 CXR images with an average pixel size of 3000 × 3000. Within the dataset, 326 of the individuals are healthy, and the other 336 have tuberculosis. The MC dataset, obtained from the tuberculosis control program of the Montgomery County (MC), MD, Department of Health and Human Services of the United States, contains 138 CXR images with pixel sizes of 4020 × 4892 or 4892 × 4020. Eighty of these are healthy, and the other fifty-eight have tuberculosis. All three datasets were augmented to increase the size of the datasets and split into an 8:1:1 ratio for training, validation, and testing.

To generalize the effectiveness of the model, another dataset, the Chest X-ray dataset [11], has been used. It consists of chest radiographs and their corresponding lung mask images. This data set contains a total of 21,165 CXR images with a pixel size of 512 × 512, along with their corresponding masks. A total of 10,000 samples were randomly selected as the dataset for the model, taking into account the 8:1:1 ratio. Of these, 8000 samples were designated as the training set, 1000 samples as the validation set, and 1000 samples as the test set.

### 3.2. Data Preprocessing and Augmentation

**Figure 2:**
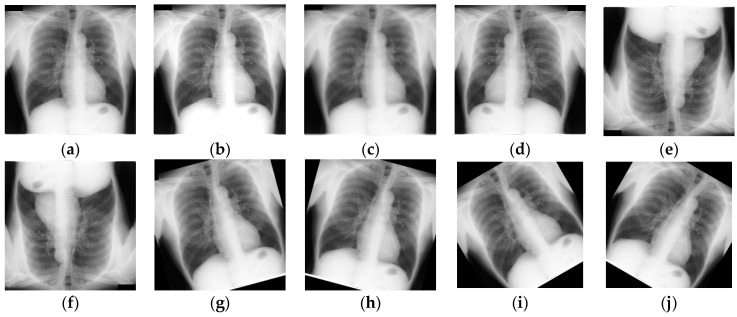
Data augmentation: (**a**) original image, (**b**) brightness adjust, (**c**) Gaussian blur, (**d**) horizontal flip, (**e**) vertical flip, (**f**) horizontal–vertical flip, (**g**) 15-degree anti-clockwise rotation, (**h**) 15-degree clockwise rotation, (**i**) 30-degree anti-clockwise rotation, (**j**) 30-degree clockwise rotation.

The preprocessing included the application of a uniform scaling procedure due to the varying sizes of the source images in the dataset. Every image was resized to 256 × 256 pixels. Since the images typically show a wide range of intensity levels, normalization was used to convert the scale from 0–255 to 0–1 by dividing the pixel values by 255, helping to normalize the images. At the same time, for the JSRT, MC, and SZ datasets, morphological changes such as rotations at different angles (−15, 15, −30, 30, −45, 45, −90, 90), vertical flipping, horizontal flipping, and flipping both axes together were used to expand the dataset, increasing the dataset from 138 to 9304 images. This augmentation helps ensure the durability and performance of the training model. Some of the data augmentation techniques are shown in Figure 2. Table 1 demonstrates the data distribution of each class.

### 3.3. Proposed Architecture

Researchers have spent the last few decades working on creating different computer-aided systems that can identify a wide range of potentially fatal diseases using images obtained during medical examinations. Accurate diagnosis is made easier by precise segmentation, which aids in the identification and delineation of anatomical structures and diseased locations. 

**Figure 3:**
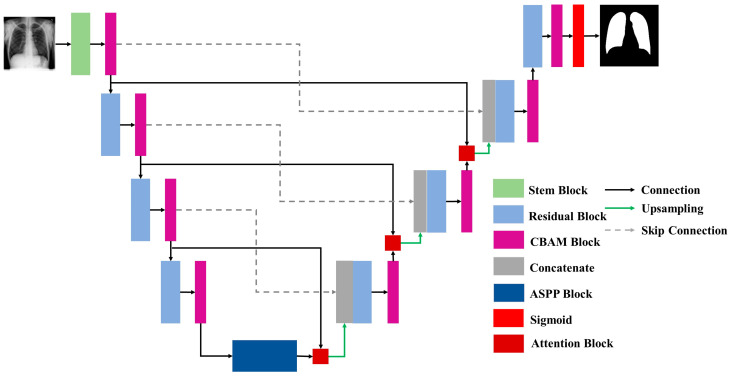
The proposed model visualization for CXR segmentation.

**Figure 4:**
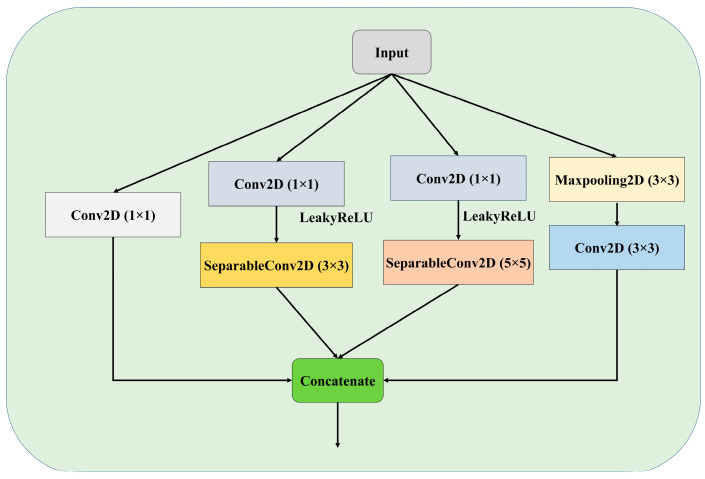
Stem block used in the proposed model.

To segment lung X-ray pictures, this work presents a modified residual U-Net architecture. Following the preprocessing stage, an architecture was constructed to extract the features from the images. In Figure 3, the proposed architecture can be seen. Figure 4 represents the stem block used in the proposed architecture. The stem block function constructs a sophisticated convolutional block that encompasses several concurrent paths, including convolutions, batch normalization, activations, and pooling. It then merges the output of these paths to augment the feature representation. The utilization of the CBAM module after the stem block further enhances the amalgamated characteristics by employing attention techniques. The proposed model is an enhanced version of the traditional U-Net architecture, which combines the strengths of U-Net with residual learning to improve training and performance. Instead of using simple convolutional layers, the proposed model replaces them with residual blocks, which improve the network’s learning efficiency by incorporating skip connections within the blocks. These connections allow the model to learn identity mappings more effectively and mitigate issues such as vanishing gradients, particularly in deeper networks. 

To further enhance feature representation, CBAM is applied after every residual block. CBAM sequentially applies channel attention and spatial attention to refine feature maps by focusing on the most informative parts of the input. This integration boosts the model’s ability to emphasize relevant features while suppressing irrelevant or noisy ones, resulting in improved segmentation performance and better generalization. The inclusion of an ASPP block is utilized as a bridge between the encoder and decoder. The ASPP block captures multi-scale contextual information by applying atrous convolutions with varying dilation rates, effectively enlarging the receptive field without increasing resolution loss. Additionally, an attention block is introduced before each upsampling step in the decoder. This attention block focuses on the most critical regions by modulating encoder features with decoder features. The output is then upsampled and concatenated with the corresponding encoder features via skip connections. Finally, the final output of the network passes through a sigmoid activation function, which transforms the logits into a probability map, producing pixel-wise probabilities between 0 and 1. This step is essential for binary segmentation tasks, as it allows for a clear delineation of foreground and background regions.

**Figure 5:**
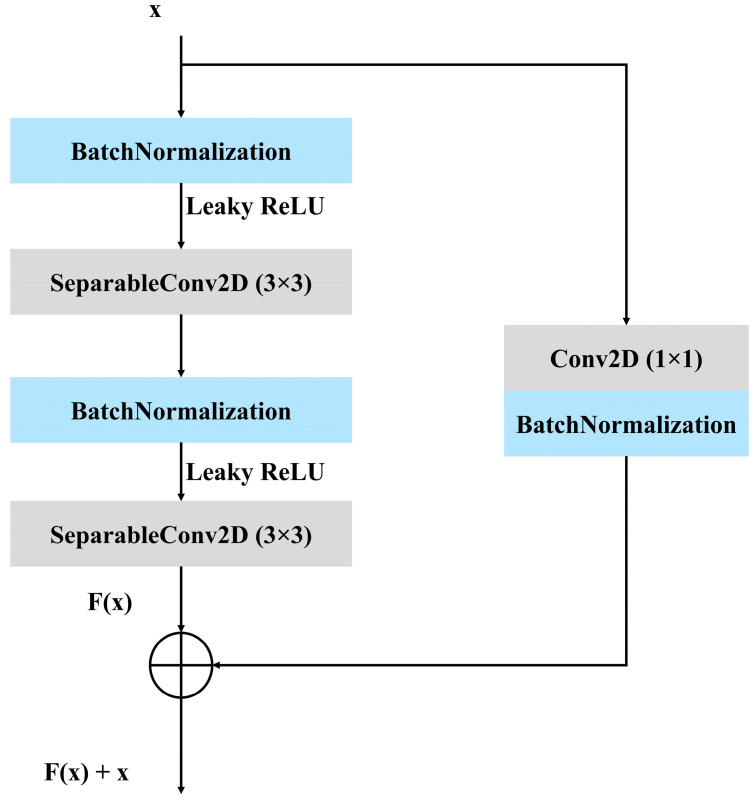
Residual block used in the proposed model.

In Figure 5, it can be seen that batch normalization, convolutional layers, and the leaky rectified linear unit (LeakyReLU) activation function combine to form the residual block. For the residual path, a 3 × 3 SeparableConv2D is used with the LeakyReLU activation function. For the skip connection, a 1 × 1 Conv2D and batch normalization are used. For downsampling, a 2 × 2 stride convolutional layers are deployed.

#### 3.3.1. Convolutional Block Attention Module (CBAM)

**Figure 6:**
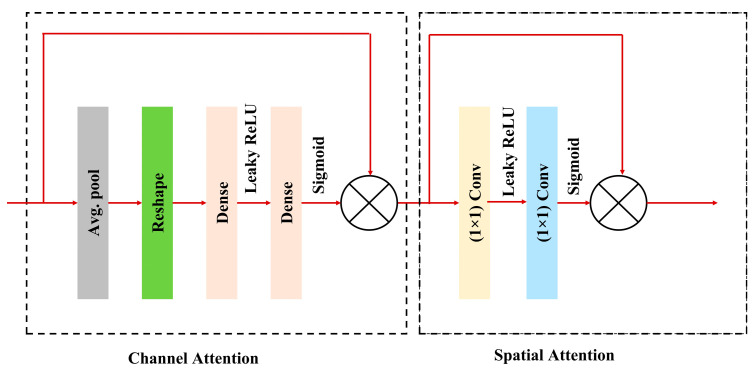
CBAM block used in the proposed model.

Any convolutional neural network architecture can incorporate the effective and lightweight CBAM attention module, which can be trained according to the base network throughout its entirety [25]. The CBAM helps the network learn more robust and discriminative features by focusing on important channels and spatial regions. The most significant advantage of this block is that it can be integrated into any kind of CNN architecture very easily, without requiring any modification. Moreover, it introduces minimal computational overhead compared to the performance improvements it provides. The CBAM used in the proposed model is shown in Figure 6. The working procedure of the CBAM block can be understood from the following mathematical expressions:

(1) $$z_{c}=\frac{1}{H\times W}\sum_{i=1}^{H}\sum_{j=1}^{W}F_{ijc}$$

where *z_c_* symbolizes the global average pooling for the channel attention. The input feature map is represented by *F_ijc_*, its height and width are represented by *H* and *W*, respectively, and its channel index is represented by *c*.

(2) $$s_{c}=\sigma \left(W_{2}\cdot F\left(W_{1}\cdot z_{c}\right)\right)$$

where *s_c_* is the output of the fully connected layers. Here, *σ* represents the sigmoid activation function, *W_1_* and *W_2_* are the weights of two fully connected layers, respectively, and *F* is the LeakyReLU activation function.

(3) $$U_{channel}=U⊙s$$

Here, *U_channel_* represents the channel attention map, ⊙ symbolizes the element-wise multiplication, and *s* is broadcast to match the dimension of *U*.

(4) $$V_{1}=F\left(W_{3}\cdot U_{channel}\right)$$

(5) $$M_{s}=\sigma \left(W_{4}\cdot V_{1}\right)$$

Here, *W_3_* and *W_4_* are the convolutional kernels representing the convolution, and *V_1_* is the intermediate feature map obtained after the first convolution and the LeakyReLU activation function. *M_s_* is the spatial attention map. The general CBAM block equation, which combines the channel and spatial attention mechanisms, can be summarized as follows:

(6) $$F_{CBAM}=U_{channel}⊙M_{s}$$

Equation (7) describes the process of applying channel attention first and then spatial attention to the input feature map *X*.

#### 3.3.2. Atrous Spatial Pyramidal Pooling

**Figure 7:**
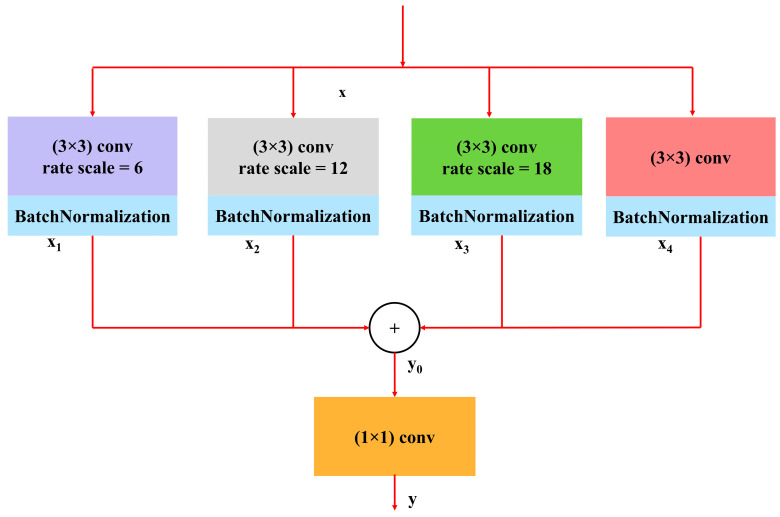
ASPP block used in the proposed model.

The idea of ASPP originated from spatial pyramid pooling [26], which effectively re-samples features at various scales. Contextual data are recorded in ASPP on several scales, and the input feature map is fused by combining numerous parallel atrous convolutions at varying rates [27,28]. ASPP enhances the network’s ability to understand the context within the image by capturing features at different scales. In the proposed model, the ASPP block is used as the bridge between the encoder and decoder sides. Figure 7 shows the ASPP block diagram used in the proposed model. The following mathematical expressions explain the ASPP block operation more clearly:

(7) $$x_{i}=BN\left(Conv_{3\times 3}\left(x,D_{j}\right)\right)$$

Here, *BN* is batch normalization, *X* is the input feature map, and *x_i_* is the output after applying a 3 × 3 convolution with dilation rate *D_j_*. For the proposed model, *D_j_* = 1, 6, 12, 18 was used. The convolution outputs with different dilation rates are combined by adding elements in a way that allows for the combined feature map to pass through a 1 × 1 convolution to produce the final output.

(8) $$y=Conv_{1\times 1}\left(\sum_{i=1}^{4}x_{i}\right)$$

Hybrid models in deep learning integrate multiple architectural components to enhance segmentation accuracy, generalizability, and computational efficiency. In medical image segmentation, particularly lung segmentation, hybrid approaches combine the strengths of convolutional neural networks (CNNs), attention mechanisms, residual learning, and multi-scale feature extraction to improve performance. For instance, TransCotANet [8] integrates a CotA module within a U-Net framework, leveraging global feature aggregation to enhance segmentation accuracy, but faces computational complexity challenges. Similarly, Pix2pix-GAN [9] employs generative adversarial networks (GANs) to refine segmentation masks, effectively addressing anatomical variations but suffering from dataset constraints and generalization issues. FAT-Net [10] combines convolutional networks with an auxiliary transformer branch, improving global contextual understanding while maintaining efficiency. 

Hybrid architectures like VAEL-Unet [11] introduce lightweight encoding with convolutional block attention modules (CBAM) to enhance feature selection while minimizing parameter count, which aligns with the proposed model design principles. ASPP-based models, such as DeepLabV3+ [17], improve segmentation by capturing multi-scale contextual information through atrous convolutions, significantly enhancing performance in complex medical images. Furthermore, AMRU++ [13] integrates multi-residual blocks with attention mechanisms, improving segmentation accuracy in diseased lungs but at the cost of increased computational demands. 

The proposed model builds upon these hybrid approaches by incorporating residual learning, CBAM, and ASPP blocks in a lightweight U-Net architecture. The residual connections mitigate vanishing gradients and enhance feature propagation, while the CBAM attention module refines spatial and channel-wise feature selection, boosting segmentation precision. The ASPP block further strengthens multi-scale feature extraction, allowing the model to capture diverse lung structures effectively. Compared to prior works, this proposed model achieves superior segmentation performance with a significantly reduced computational footprint, making it more practical for real-time applications in clinical settings. By leveraging hybrid deep learning techniques, this approach ensures improved segmentation robustness, efficiency, and adaptability across various lung imaging datasets.

## 4. Result Analysis

In this section, the experimental setup and results of this research are described in detail.

### 4.1. Hyperparameter Selection and Experimental Configuration

To achieve a high level of accuracy with the proposed model, it is necessary to carefully adjust and fine-tune its hyperparameters. The values of these hyperparameters are presented in Table 2. The hyperparameters were optimized using a combination of empirical analysis and systematic tuning to achieve the best balance between model accuracy and computational efficiency. For learning rate optimization, values in the range of 0.0001–0.01 were tested. A learning rate of 0.0001 resulted in slow convergence, requiring more epochs to reach optimal performance, whereas a value of 0.01 caused instability, leading to fluctuating loss values and difficulty in convergence. The optimal balance was found at 0.001, which ensured stable convergence while avoiding vanishing or exploding gradients. 

For batch size selection, experiments were conducted with values of 16, 32, and 64. A batch size of 16 led to slower training due to limited parallelization and resulted in higher variance in model updates, which affected stability. Conversely, a batch size of 64 required more memory and occasionally led to suboptimal convergence, as larger batches can smooth gradients excessively and reduce generalization capability. A batch size of 32 was manually selected as the best trade-off, ensuring stable updates, efficient GPU utilization, and improved generalization. To analyze the impact of loss functions and optimizers, an additional study was conducted by varying these parameters. Adam, RMSprop, and SGD optimizers were compared, with Adam providing the best trade-off between convergence speed and accuracy. These optimizations collectively enhanced model robustness, segmentation accuracy, and computational efficiency. Research evidence suggests that the Adam optimizer outperforms other widely used optimizers in terms of achieving higher rates of learning. In addition, batch normalization layers were included to accelerate model training. In the proposed model, the different hybrid combinations of loss functions were deployed. The compound losses can be explained by the following expression:

(9) $$L_{total}=\alpha L_{Dice}+\beta L_{BCE}+\gamma L_{Focal}$$

where α, β and γ control the contribution of each term. For simplicity, equal weights have been assigned to each component in the compound loss functions. Specifically, the weighting parameters were set to α=β=γ=1 in all combinations, meaning each loss function contributed equally to the total loss. It was found that the Dice loss produced the best result for the proposed model. Therefore, the Dice loss was used as the loss function for the proposed model.

The balance between memory consumption and training speed influences practical decisions about batch size. In this investigation, a batch size of 32 was used for normalization. Following numerous experiments conducted with training data from the combined dataset, the remaining hyperparameter values were selected somewhat arbitrarily. A trial-and-error methodology was employed to iteratively fine-tune the hyperparameters for optimal performance. Table 3 presents the system configuration used in this study.

### 4.2. Experimental Results

Various tests have been carried out to determine the proposed segmentation model and to evaluate the impacts of various adjustments. These experiments include the following:

- The U-Net model, acting as the original benchmark for semantic segmentation;
- The residual U-Net model, serving as the initial standard for the proposed model;
- Residual U-Net with attention-guided skip connections, without adding CBAM to the network;
- Proposed model with CBAM added after the residual block, with RELU activation;
- Proposed model with CBAM added after the residual block, with LeakyReLU activation.

The results in Table 4 show the comparative analysis of each modification stated above for the JSRT dataset. The U-Net model achieved a Dice score of 98.34%, an accuracy of 99.03%, an IoU of 96.74%, a precision of 98.32%, and recall and specificity of 98.38% and 99.22%, respectively, showing a moderate result. Upgrading the network to residual U-Net slightly increases the results, achieving a Dice score of 98.39%, and an IoU of 96.83%, with improvements across all other metrics compared to the U-Net. Adding an attention mechanism to the skip connections further improves the results, achieving a higher Dice score than both the U-Net and residual U-Net models. This model achieved a 98.44% Dice score, with an IoU of 96.94%, and all other metrics above 98%. 

The proposed model with CBAM integrated inside the network with the RELU activation function improves the overall result, achieving a Dice score of 98.52%, indicating the efficiency of the network in segmenting lung areas. Using the LeakyReLU as the activation function further improves the model’s efficiency in lung segmentation, achieving the highest results across all metrics. This modification achieved a Dice score of 98.72%, an accuracy of 99.24%, an IoU of 97.48%, and precision, recall, and specificity of 98.76%, 98.68%, and 99.46%, respectively. From the table, it can be seen that the addition of CBAM has improved the performance, while the use of LeakyReLU further enhanced the accuracy and the quality of segmentation. CBAM increased the ability of a model to represent information by prioritizing significant characteristics in both the channel and spatial dimensions. Consequently, this results in improved segmentation performance. Based on Table 4, Table 5 and Table 6, it is evident that the proposed model exhibits higher Dice scores and IoU values, indicating a higher level of precision in overlapping the predicted mask with the ground truth. The combination of CBAM applied after residual blocks and the use of LeakyReLU activation improved overall performance by refining feature representation and capturing both global and local spatial dependencies for accurate segmentation. 

**Figure 8:**
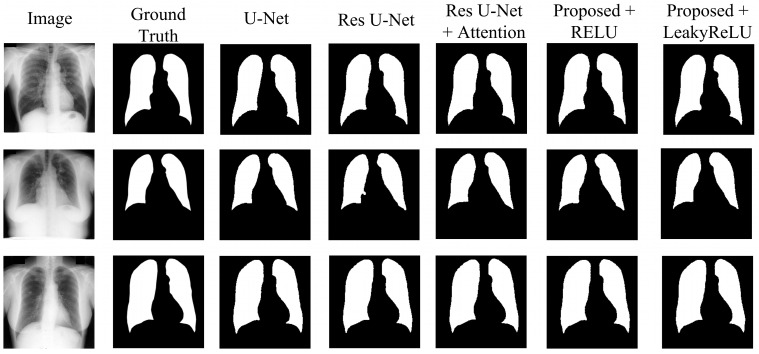
Segmentation quality comparison of the JSRT dataset.

**Figure 9:**
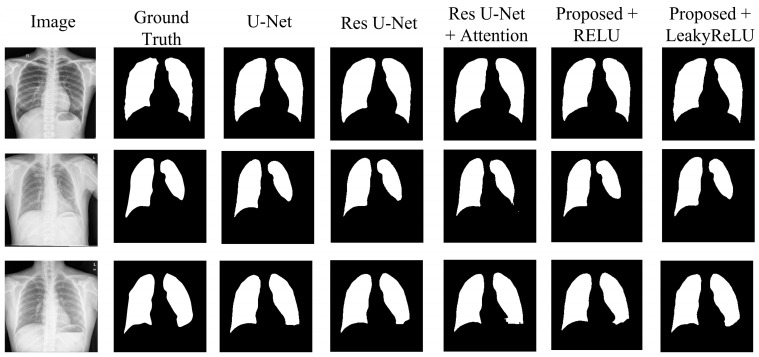
Segmentation quality comparison of the SZ dataset.

**Figure 10:**
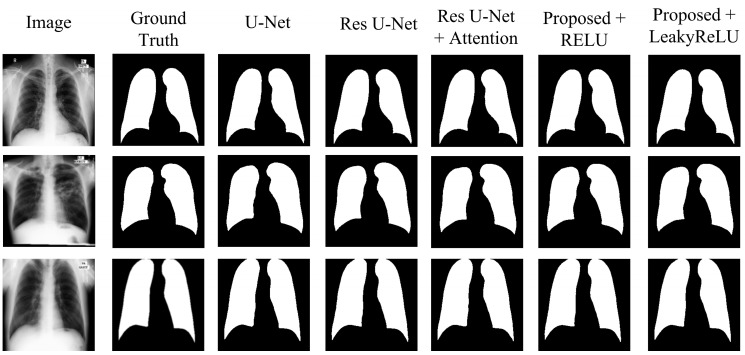
Segmentation quality comparison of the MC dataset.

To ensure consistency, the same set of hyperparameters was utilized to evaluate the JSRT, SZ, and MC datasets. Results in Table 5 and Table 6 validate the performance of the proposed model for the SZ and MC datasets, respectively. Table 5 presents the results obtained from the SZ dataset. From the table, it can be seen that the proposed model also achieved the highest results across all metrics compared to the other models. With the SZ dataset, the proposed model achieved a Dice score of 97.49% and a mean IoU of 95.13%, showcasing its generalizability across different datasets. Table 6 shows the segmentation performance on the MC dataset. The proposed model achieved the highest score in this case as well, achieving a Dice score of 99.08% and a mean IoU of 98.18%, demonstrating the strong generalization capability of the model. Figure 8, Figure 9 and Figure 10 visually illustrate the improvements and accuracy gains achieved through the modifications made to the proposed model across the JSRT, SZ, and MC datasets, respectively. Through conducting these trials and analyses, the performance of the segmentation model was improved step by step. This involved finding the most impactful adjustments, such as incorporating CBAM blocks and using the LeakyReLU activation function rather than RELU. These enhancements greatly improved the accuracy and scalability of the proposed model, establishing it as a sophisticated solution for segmenting lung regions from CXR images.

Table 7 showcases a comparison of total parameters, trainable parameters, and model size across different models. U-Net, serving as a benchmark model, has the second-lowest total number of parameters at 3.27 M (12.5 MB). The residual U-Net introduces an increase in total parameters, indicating a more complex network than U-Net, with 5.06 million (19.32 MB) parameters. Adding an attention mechanism to the skip connection between the encoder and decoder further increases the total parameters to 5.86 million (22.35 MB), indicating that attention mechanisms increase the complexity of the model. However, the proposed model has the least number of parameters—3.24 million (12.31 MB)—making it the most lightweight among the models mentioned. CBAM is specifically engineered to have a low weight, resulting in a minimum computational burden while potentially improving performance. From the table, it is evident that adding CBAM to the network significantly reduced the parameter size while achieving the best performance among the models.

**Figure 11:**
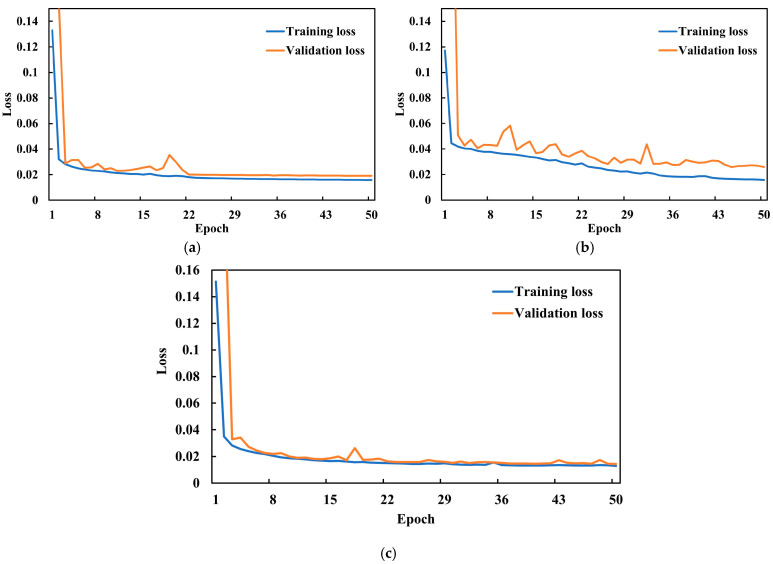
Model loss curves: (**a**) JSRT dataset, (**b**) SZ dataset, (**c**) MC dataset.

The training and validation loss curves for the JSRT, SZ, and MC datasets are shown in Figure 11. Each plot demonstrates that the training and validation losses progressively decrease as the number of epochs increases, eventually stabilizing. Additionally, the losses decrease to below 0.02 for the JSRT and SZ datasets.

### 4.3. Experimental Results on the Chest X-Ray Dataset for External Validation

**Figure 12:**
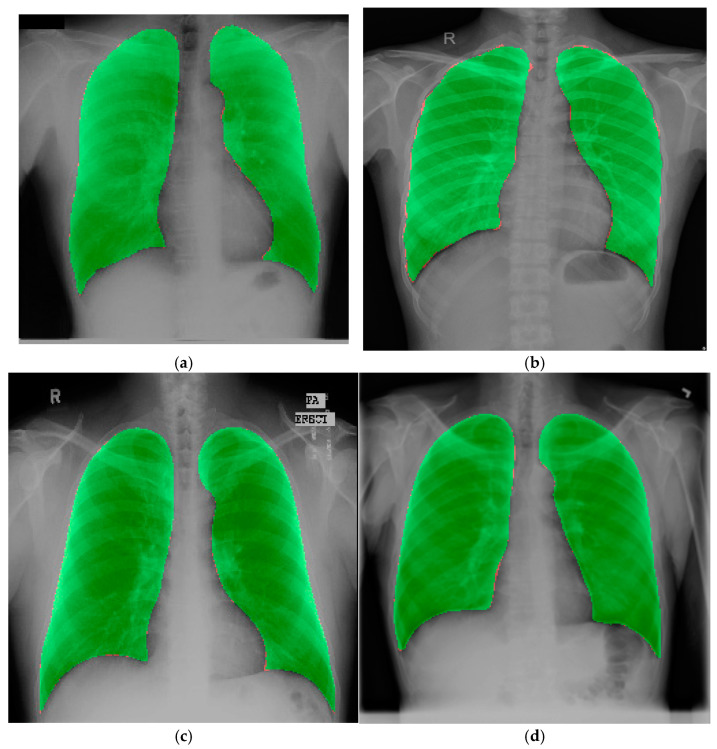
Original mask and proposed model’s predicted mask overlay: (**a**) JSRT dataset, (**b**) SZ dataset, (**c**) MC dataset, (**d**) Chest X-ray dataset. (The red spot represents the original mask area, and the green spot represents the predicted mask area).

To validate the effectiveness of the proposed model, another dataset was used [11] for external validation. In order to assess the accuracy of the proposed model, a 5-fold cross-validation test was conducted using the Chest X-ray dataset. Table 8 displays the outcomes of the 5-fold cross-validation test. The table shows consistent performance across all folds, with minimal variation in metrics such as accuracy and Dice score, with a very low standard deviation indicating the model’s stability and reliability. Cross-validation helps identify whether the model generalizes well to unseen data by testing it on diverse subsets of the dataset, reducing the risk of overfitting. The low standard deviations across metrics highlight the statistical consistency of the results, further validating the robustness of the chosen loss function and model architecture. This approach ensures that the reported performance of the proposed model is representative of its true capability. Figure 12 represents original mask and proposed model’s predicted mask overlay.

### 4.4. Additional Experiments

A series of experiments were conducted using various loss combinations, and the consolidated results are shown in Table 9. This table highlights the performance metrics of the segmentation model evaluated using various loss functions. Each loss function or combination yields slightly different outcomes across key metrics such as Dice score, IoU, recall, precision, and specificity. Notably, Dice loss achieves the best Dice score and IoU, while focal loss shows weaker performance in Dice and IoU but demonstrates strong precision. These variations underline the importance of testing the model using different loss functions, as each one optimizes different aspects of model performance. For segmentation tasks, where pixel-level accuracy is crucial, the choice of loss function directly impacts the balance between false positives and false negatives, influencing the model’s generalizability and application-specific reliability. After analyzing Table 9 and Table 10, it can be seen that the Dice loss is the most efficient loss function, and Adam is the best optimizer for the proposed model. Table 11 shows statistically significant differences (*p* < 0.05) in accuracy, Dice score, and IoU for I, II, III, and IV.

## 5. Discussion

### 5.1. Comparative Analysis

Table 12 provides a comparative evaluation of the proposed model against various state-of-the-art (SOTA) models, demonstrating how the novel Residual U-Net with CBAM and LeakyReLU overcomes key limitations identified in previous studies. To evaluate the effectiveness of the proposed model, the segmentation performance is compared with multiple SOTA models using the same datasets: JSRT, SZ, MC, and Chest X-ray datasets. One of the key advantages of the proposed model is its ability to achieve higher segmentation accuracy while maintaining the lowest parameter count among all compared models. Most SOTA models exhibit high performance but at the cost of increased computational complexity, requiring a significantly larger number of parameters and extensive training resources. In contrast, the proposed model, which integrates Residual U-Net with CBAM and ASPP, achieves a balance between accuracy and efficiency. For instance, models such as TransCotANet (Xu et al. [8]) and FAT-Net (Khorasani et al. [10]) rely on complex transformer-based architectures that enhance segmentation accuracy but suffer from high computational costs. The proposed model, however, leverages CBAM to selectively enhance important spatial and channel-wise features, thereby improving segmentation accuracy while keeping the model lightweight. This results in a higher Dice score and IoU compared to these models, with significantly fewer parameters. Similarly, AMRU++ (Alam et al. [13]) and U-Net++ (Gite et al. [14]) demonstrate strong segmentation performances but require over 10 million parameters, making them less suitable for real-time applications. In contrast, the proposed model maintains only 3.24 million parameters, making it one of the lightest architectures while achieving superior segmentation performance. 

A critical factor influencing segmentation quality is the ability to handle diverse image variations and pathological abnormalities. Models such as Pix2pix-GAN (Sharmin et al. [9]) have shown promising results in generating high-quality segmentations but struggle with generalization due to dataset constraints. The proposed model addresses this limitation by undergoing external validation on the Chest X-ray dataset, where it consistently achieved high accuracy across all folds, as shown in Table 8. Furthermore, in DeepLabV3+ (Hasan et al. [17]), the use of atrous convolutions enhances feature extraction, but its computational complexity is significantly higher than the proposed model. By incorporating ASPP in a lightweight U-Net framework, the proposed model retains the benefits of multi-scale feature extraction while avoiding the overhead of heavier architectures. Lastly, comparing the total number of parameters across models highlights the advantage of the proposed model in achieving high segmentation accuracy with minimal computational cost, making it more suitable for real-time applications and deployment in resource-constrained environments. 

In summary, the proposed model surpasses existing SOTA models by offering a lighter architecture with comparable or superior accuracy, improved generalization across datasets, and significantly reduced computational complexity, making it a practical choice for lung segmentation in medical imaging applications. Models like TransCotANet [8], FAT-Net [10], and EfficientNet-B4 with Residual Blocks and LeakyReLU [29] are inherently more complex and computationally demanding compared to the proposed framework. TransCotANet integrates a transformer-based architecture with multidimensional global feature aggregation, significantly increasing the number of parameters due to self-attention mechanisms and additional feature fusion layers. Transformer-based models generally require extensive matrix multiplications and sequential token processing, leading to higher computational costs and memory consumption. FAT-Net also employs transformers through an auxiliary transformer branch designed to enhance global contextual understanding. While this improves segmentation performance, it substantially increases the number of trainable parameters due to additional transformer layers and the fusion of multi-scale features. The computational complexity of FAT-Net is further elevated by its dual encoder–decoder structure, which processes feature representations at multiple levels, leading to higher memory requirements and longer inference times. EfficientNet-B4 with Residual Blocks and LeakyReLU follows a scaling approach that increases network depth, width, and resolution to improve performance. However, using EfficientNet as an encoder contributes to a higher parameter count and computational overhead. In contrast, the proposed model employs a lightweight Residual U-Net structure enhanced with CBAM and ASPP, which selectively refines spatial and channel features without introducing excessive parameters. By focusing on efficient attention mechanisms rather than transformer-based feature aggregation or deep scaling strategies, the proposed model achieves high segmentation accuracy while maintaining a significantly lower parameter count, making it more suitable for real-time applications and resource-constrained environments.

### 5.2. Strengths, Limitations, and Future Scope

Predicting accurate lung segmented mask images from noisy images is a challenging task. However, this study successfully addresses these challenges by employing a novel DL model that outperforms SOTA models. The proposed model leverages CBAM applied to the residual blocks, significantly enhancing accuracy by dynamically adjusting the weights of feature maps based on global context and channel-wise information. This allows the network to focus on informative spatial regions while channel attention selectively emphasizes important features, improving segmentation performance. Additionally, despite having the lowest number of parameters compared to all other SOTA models, the proposed model achieves superior results while maintaining an optimal balance between accuracy and complexity. Its lightweight architecture makes it highly suitable for deployment on edge devices, enabling real-time lung segmentation in resource-constrained environments. Another key advantage of this study is the external validation performed on an independent dataset—a crucial step that most SOTA models have not accomplished. This validation demonstrates the robustness and generalizability of the proposed model, further establishing its effectiveness over existing methods. The use of the LeakyReLU activation function further strengthens the model by preventing neuron “death,” mitigating the vanishing gradient problem, and ensuring more accurate segmentation. Despite its strengths, the model has slight limitations in recall and precision, which can be further optimized in future research. Enhancing these metrics could make the model even more effective for real-world clinical applications.

### 5.3. Clinical Significance

To distinguish lung features from medical pictures such as CT scans or X-rays, lung segmentation is an essential step in medical image analysis. Pneumonia monitoring, disease diagnosis, and treatment planning are just a few of the healthcare applications that depend on accurate lung segmentation. Due to overlapping structures such as bones, individual differences in lung morphology, pathological opacities, foreign objects that obstruct lung fields, and minor abnormalities that are often overlooked, it can be difficult to identify lung disorders from X-ray images [31]. By precisely isolating lung regions, improving abnormality detection, standardizing the area of interest for more consistent analysis, enabling automated detection and diagnosis through DL models, and lessening the impact of artifacts by excluding non-lung areas, lung segmentation can help mitigate these problems. By precisely predicting lung mask images, radiologists can more effectively identify, detect early, and manage high-risk lung diseases such as lung cancer, pneumonia, tuberculosis, emphysema, and COPD. Early detection of these diseases can lead to improved treatment and chances of recovery. The clinical significance of this research lies in helping radiologists by predicting the lung mask images to identify the early symptoms of high-risk lung diseases.

## 6. Conclusions

In conclusion, lung segmentation is crucial in medical imaging, providing substantial advantages in the identification, treatment, and study of lung disorders. The ongoing development of segmentation techniques, namely the use of advanced deep learning models, holds the potential to improve the accuracy and usefulness of lung segmentation in clinical settings. In this study, Residual U-Net was used as the baseline model, and the CBAM attention module was integrated into the residual blocks to create the proposed model. The ASPP block was also used as the bridge between the encoder and decoder, and attention mechanisms were used in the skip connections. These combined modifications improved the lung segmentation quality of the proposed model while maintaining the lowest number of parameters. The proposed model was evaluated across three benchmark datasets: JSRT, SZ, and MC. The model achieved Dice scores of 98.72%, 97.49%, and 99.08% on the JSRT, SZ, and MC datasets, respectively, outperforming all the previous models. Furthermore, the model was externally evaluated using the Chest X-ray dataset, resulting in the highest Dice score of 98.93%. These results indicate that the proposed model is a resilient and effective model that performs well in many datasets. In the future, a semi-supervised model can be developed to further enhance the lung segmentation process. The use of semi-supervised learning has great potential to improve lung segmentation in medical imaging.

## Figures and Tables

**Table 1:** Data distribution in each dataset for training, validation, and testing.

Dataset	Original Image Samples	Training Images	Augmented Training Images	Validation Images	Testing Images
JSRT	247	198	8000	25	25
SZ	662	530	8000	66	66
MC	138	110	8000	14	14

**Table 2:** Hyperparameter configurations of the proposed framework.

Hyperparameters	Value
Batch size	32
LeakyReLU negative slope	0.01
Optimizer	Adam
Initial learning rate	0.001
Epochs	50
LR reduce factor	0.1
Loss function	Dice Loss

**Table 3:** System configurations of the proposed framework.

Tools	Configuration
Programming Language	Python
Backend	Keras with TensorFlow
Disk Space	78.2 GB
GPU RAM	15 GB
GPU	Nvidia Tesla T4
System RAM	12.72 GB
Operating system	windows 10
Input	Lung images
Input Size	256 × 256

**Table 4:** Segmentation performance of the JSRT dataset.

Model	Accuracy	Dice	IoU	Recall	Precision	Specificity
I.	99.03	98.34	96.74	98.38	98.32	99.22
II.	99.04	98.39	96.83	98.39	98.39	99.30
III.	99.06	98.44	96.94	98.42	98.47	99.34
IV.	99.11	98.51	97.06	98.50	98.52	99.36
V.	99.24	98.72	97.48	98.68	98.76	99.46

**Table 5:** Segmentation performance of the SZ dataset.

Model	Accuracy	Dice	IoU	Recall	Precision	Specificity
I.	98.33	96.32	93.00	95.69	97.13	99.17
II.	98.47	96.65	93.56	96.83	96.56	98.99
III.	98.37	96.41	93.15	95.79	97.17	99.18
IV.	98.83	97.41	94.98	97.12	97.74	99.33
V.	98.88	97.49	95.13	97.29	98.04	99.42

**Table 6:** Segmentation performance of the MC dataset.

Model	Accuracy	Dice	IoU	Recall	Precision	Specificity
I.	98.15	96.27	92.93	96.17	96.55	98.86
II.	98.29	96.57	93.47	96.11	97.17	99.07
III.	98.22	96.42	93.20	95.87	97.13	99.07
IV.	98.26	96.51	93.36	96.05	97.12	99.06
V.	99.56	99.08	98.18	99.34	98.83	99.62

**Table 7:** Parameter and size comparison between different models.

Models	Total Parameters (Millions)	Trainable Parameters (Millions)	Size (MB)
U-Net	3.27	3.27	12.50
Res U-Net	5.06	5.05	19.32
Res U-Net+ Attention	5.86	5.85	22.35
Proposed	3.24	3.23	12.37

**Table 8:** Segmentation performance on the Chest X-ray dataset for external validation using 5-fold cross-validation (trained on the MC dataset).

5-Fold CV	Accuracy	Dice	IoU	Recall	Precision	Specificity
Fold-1	99.490	98.902	97.893	98.824	99.027	99.703
Fold-2	99.509	98.951	97.975	98.876	99.067	99.714
Fold-3	99.510	98.931	97.950	98.835	99.086	99.727
Fold-4	99.467	98.842	97.806	98.721	99.044	99.712
Fold-5	99.490	98.899	97.892	98.834	99.025	99.710
Mean	99.493	98.905	97.903	98.818	99.050	99.713
Std. Dev	0.018	0.041	0.065	0.058	0.026	0.008

**Table 9:** Segmentation performance over different linear combinations of loss functions. (MC dataset).

Loss	Accuracy	Dice	IoU	Recall	Precision	Specificity
BCE loss	99.45	98.81	97.73	98.659	99.030	99.723
Dice loss	99.56	99.08	98.18	99.34	98.83	99.62
Focal loss	98.612	98.641	96.381	98.554	99.177	98.535
BCE + Dice	99.480	98.864	97.845	98.824	98.978	99.696
BCE + Focal	99.409	98.721	97.538	98.891	98.604	99.575
Dice + Focal	99.464	98.848	97.804	98.865	98.899	99.659
BCE + Dice + Focal	99.414	98.721	97.577	98.721	98.812	99.645

**Table 10:** Segmentation performance across different optimizers with Dice loss functions. (MC dataset).

Loss	Accuracy	Dice	IoU	Recall	Precision	Specificity
Adam	99.45	98.81	97.73	98.659	99.030	99.723
RMSprop	97.32	96.85	94.56	96.21	97.45	98.65
SGD	94.27	93.89	91.12	93.45	94.12	96.32

**Table 11:** *p*-values from the Mann–Whitney U test for comparing the proposed model (v) with other combinations for the MC dataset (A *p*-value greater than 0.05 indicates that the difference between the models is not statistically significant.).

Model	*p*-Value (Accuracy)	*p*-Value (Dice)	*p*-Value (IoU)
I.	0.032	0.041	0.028
II.	0.015	0.048	0.039
III.	0.035	0.036	0.027
IV.	0.022	0.033	0.018

**Table 12:** Comparison of segmentation performance of the proposed model with different state-of-the-art (SOTA) models.

Reference	Model	Dataset Used	Accuracy	Dice	IoU	No. of Total Paramters (M)
Xu et al. [8]	TransCotANet	JSRT SZ MC	99.14 98.46 98.91	99.03 97.66 98.02	98.76 94.41 97.89	-
Sharmin et al. [9]	Pix2pix-GAN	SZ MC	95.87 98.25	- 98.05	-	11.5
Khorasani et al. [10]	FAT-Net	SZ, MC (Merged)	98.12	96.10	-	-
Hao et al. [11]	VAEL-Unet	Chest X-ray	97.69	- -	93.65	1.1
Alam et al. [13]	AMRU++	MC + JSRT SZ	- -	96.28 93.38	93.46 87.97	10.65
Gite et al. [14]	U-Net++	SZ MC	98.74 97.71	97.96 96.30	95.96 92.93	36.64
Hasan et al. [17]	DeeplabV3+	SZ	97.42	96.63	93.49	11.85
Liu et al. [29]	Efficientnet-b4 encoder + Residual blocks + LeakyReLU	JSRT MC	98.55 98.94	97.92 97.82	95.73 95.55	-
Tam et al. [30]	DDRU-Net	SZ MC	98.23 99.35	94.84 98.87	90.30 97.77	64.65
Din et al. [22]	CXR-Seg	MC SZ	98.77 96.69	97.76 96.32	95.63 92.97	5.98
Proposed	Residual U-Net with CBAM+ LeakyReLU	JSRT SZ MC Chest X-ray	99.24 98.88 99.56 99.51	98.72 97.49 99.08 98.93	97.48 95.13 98.18 97.95	3.24

## Data Availability

The data are contained within this article.
